# Contemporary cinemeducation: Transdisciplinary exchange at Locarno Film Festival

**DOI:** 10.3205/zma001852

**Published:** 2026-06-15

**Authors:** Mike Rueb, Stefano Knuchel, Justine Knuchel, Kevin L. Lee, Martin R. Fischer

**Affiliations:** 1 LMU Munich, LMU University Hospital, Institute of Medical Education, Munich, Germany; 2 Charité – Universitätsmedizin Berlin, Charité Campus Mitte, Department of Psychiatry and Neurosciences, Berlin, Germany; 3 Charité – Universitätsmedizin Berlin, Charité at St. Hedwig Hospital, Department of Psychiatry and Psychotherapy, Berlin, Germany; 4 Locarno Film Festival, BaseCamp, Locarno, Switzerland; 5 Università della Svizzera italiana, Locarno Film Festival Professor for the Future of Cinema and the Audiovisual Arts, Lugano, Switzerland; 6 Medical University of Vienna, Teaching Center, Vienna, Austria

**Keywords:** cinema, movie, cinemeducation, medical humanities

## Abstract

Film-based pedagogy, especially cinemeducation as a teaching methodology, has become more visible as an arts and humanities teaching format. However, many existing cinemeducation courses at medical schools use films that, while valuable in their time, may no longer reflect current societal or medical discourses. This study aimed to identify contemporary films that resonate with present-day issues in medicine, assess students’ motivation to engage with cinemeducation, and explore interest in transdisciplinary learning. For the first time, three medical students participated in BaseCamp at the Locarno Film Festival, a young talents program that fosters transdisciplinary exchange. They screened festival films with medical themes and organised a 75-minute workshop with students from film, natural sciences, arts, and medicine. The workshop focused on how students from these fields can collaborate to create transdisciplinary learning environments. The students identified seven films with medical topics in the Locarno Film Festival program. Evaluations showed that the students were highly motivated to further engage with cinemeducation and promote transdisciplinary education. The exchange generated innovative approaches, including the use of video essays for cinemeducation and producing films together with art and film students. Medical students like to exchange ideas with other cinemeducation projects worldwide, which is supported by a mean Likert score of 1.3 (n=3). A transdisciplinary medical film festival and a cinemeducation symposium could be a first step.

## Abstract (de)

Film-basierte Pädagogik, insbesondere Cinemeducation als Lehrmethode, hat als Lehrformat der Arts und Humanities zunehmend an Sichtbarkeit gewonnen. Viele bestehende Cinemeducation-Kurse an medizinischen Fakultäten greifen jedoch auf Filme zurück, die zwar zu ihrer Zeit bedeutsam waren, aber heutige gesellschaftliche oder medizinische Diskurse nicht mehr angemessen widerspiegeln. Ziel dieser Studie war es, zeitgenössische Filme zu identifizieren, die mit aktuellen Fragestellungen in der Medizin resonieren, die Motivation von Studierenden zur Auseinandersetzung mit Cinemeducation zu untersuchen sowie das Interesse an transdisziplinärem Lernen zu explorieren. Erstmals nahmen drei Medizinstudierende am BaseCamp des Locarno Film Festivals teil, einem Nachwuchsprogramm, das transdisziplinären Austausch fördert. Sie sichteten Festivalfilme mit medizinischen Themen und organisierten einen 75-minütigen Workshop mit Film-, Naturwissenschafts-, Kunst- und Medizinstudierenden. Der Workshop konzentrierte sich darauf, wie Studierende dieser Disziplinen gemeinsam transdisziplinäre Lernumgebungen gestalten können. Die Studierenden identifizierten sieben Filme mit medizinischem Bezug im Programm des Locarno Film Festivals. Die Evaluation zeigte, dass die Studierenden hoch motiviert waren, sich weiter mit Cinemeducation auseinanderzusetzen und transdisziplinäre Lehre zu fördern. Der Austausch brachte innovative Ansätze hervor, darunter den Einsatz von Videoessays für Cinemeducation sowie die gemeinsame Filmproduktion mit Kunst- und Filmstudierenden. Medizinstudierende wünschen sich zudem einen Austausch mit anderen Cinemeducation-Projekten weltweit, was durch einen mittleren Likert-Wert von 1,3 (n=3) bestätigt wurde. Ein transdisziplinäres medizinisches Filmfestival sowie ein Cinemeducation-Symposium könnten hierfür einen ersten Schritt darstellen.

## Introduction

Medical humanities are a multidisciplinary field in medical education incorporating methods from the arts, humanities, and social sciences [1]. The arts and humanities can foster a deeper understanding of patients’ experiences of illness [2]. Film-based pedagogy, especially cinemeducation as a teaching methodology, has become more visible as an arts and humanities teaching format [3]. Recently, a conceptual framework for cinemeducation was published [4]. Films provide a biopsychosocial perspective on illnesses, enriching their understanding beyond traditional clinical approaches [5]. They can help change attitudes and opinions, enhance interprofessional collaboration, and connect medical knowledge with emotional narratives [4]. However, many existing cinemeducation courses in medical school rely on outdated films. As a result, the medical discourses portrayed may no longer align with current scientific knowledge or societal practice. Interconnected and complex health issues demand close collaboration across multiple professions and disciplines [6]. Yet, medical students rarely have opportunities to learn alongside peers from other fields. Cinemeducation presents a valuable environment for such transdisciplinary learning [7]. However, the extent to which students from different disciplines are interested in cinemeducation is unclear.

The aim of this continuing transdisciplinary exchange was, first, to collect contemporary films for cinemeducation projects; second, to explore to what extent the medical humanities exchange motivates students to further engage in arts and humanities courses at their faculties; and third, to investigate if medical, film, natural sciences, and arts students are interested in learning together.

## Project description

The Locarno Film Festival is one of the most prestigious international film festivals. For the first time, three medical students were welcomed to BaseCamp, an eleven-day young talents program at the festival, as part of a medical humanities exchange. The program is an open field for experimentation, a chance to embrace new perspectives, and a free space for limitless multidisciplinary approaches [https://festivalbasecamp.ch/]. BaseCamp is a project that aims to create a community made for and by emerging talents between the ages of 18 and 30 that gives them a chance to experience the entire Locarno Film Festival program. Part of BaseCamp is BaseCamp Academy, a diverse, multidisciplinary workshop program featuring daily meetings with prominent figures in the cultural and intellectual fields.

The medical students, all involved in organising cinemeducation projects in Munich and Vienna, were tasked with screening the film festival program to identify films that could stimulate discussion on medical topics among students in future cinemeducation events.

For BaseCamp Academy, the medical students organised a 75-minute workshop. Eight film, five natural sciences, four arts, and three medical students participated in the workshop. In the first 15 minutes, the concepts of cinemeducation and medical humanities were introduced. The following 60 minutes were dedicated to an open discussion with all students, centred around the question: How can studies of the arts, film, medicine, and natural sciences converge to create a future transdisciplinary learning environment using films?

We used a mixed-methods evaluation with eleven open qualitative (see table 1 (Tab. 1)) and five quantitative questions administered on the final day of the exchange. We analysed the quantitative data descriptively, while the qualitative responses were reviewed using thematic content analysis to identify key patterns and insights.

## Results

The students identified 7 (3.1%) films on medical topics they could use in their cinemeducation courses from 225 programmed films (see table 2 (Tab. 2)). Among these films, the students particularly favoured Electric Child by Simon Jaquemet, which explores the use of artificial intelligence for rare diseases; Salve Maria by Mar Coll, which examines post-partum depression; and Akipleša by Saule Bliuvaite, which focuses on anorexia nervosa.

Medical student 3 was surprised:

*[…] by the strong interest art and film students showed in cinemeducation. Almost everyone immediately wanted to learn more about the project, and we kept receiving suggestions about films that might be suitable.*

The qualitative data revealed five themes (see table 3 (Tab. 3)). The medical students suggested using video essays for cinemeducation and producing films as part of a transdisciplinary project with art and film students during BaseCamp or in medical school.

Medical student 3 responded to the question of what he learned during the exchange:

I* have discovered a wide range of ways art can have an impact – through sound, narrative, or imagery. At the same time, a reflective process was triggered: instead of merely interpreting rationally, direct experience is an essential form of reception. Is factual, rational learning overrated, even in medicine? Perhaps effective learning arises more from being affected – as in bedside teaching or an M23 Cinema evening (cinemeducation course).*

Overall, the exchange motivated the medical students and affirmed a transdisciplinary Medical Humanities curriculum and collaboration with other cinemeducation projects (see table 4 (Tab. 4)).

The workshop participants expressed a need to discuss cinemeducation and its relevance, which left them with insufficient time to address the concept of transdisciplinary learning environments. Small group discussions could effectively explore this topic in greater depth in future workshops. Natural science students said they learned clinical narratives on biomedical issues and wanted to incorporate films into their curricula. Film and art students gained contemporary medical discourses and narratives for their films. All students indicated they could envisage creating short films on medical issues as part of a transdisciplinary team. The students wished for a deeper and more frequent exchange with art and film students to further explore the intersections.

## Discussion

Watching films at film festivals enables medical students to access films with relevant medical content more rapidly for their studies, which they can more easily integrate into cinemeducation courses. This approach fosters learning through engagement with current societal medical discourses.

Exposure to current films at film festivals motivated students to find films for contemporary cinemeducation. It is possible that the program included additional films addressing medical themes, as the students were not able to watch all of the festival’s films. The transdisciplinary dialogues with other young professionals encouraged them to engage more actively and establish cinemeducation courses at their universities. It also led to innovative ideas such as using video essays for cinemeducation or producing (short) films together with art and film students to engage more creatively with patient perspectives. The transdisciplinary exchange showcased the relevance of cinemeducation across the arts, film, natural sciences, and medicine. The workshop demonstrated a strong transdisciplinary interest in medicine and highlighted cinemeducation as an effective concept for enabling a transdisciplinary learning exchange.

Future research should focus on more robust study designs and collecting data from transdisciplinary students, ideally through larger, multicentre studies with longitudinal or mixed method approaches to capture changes over time and contextual factors.

## Conclusion

In conclusion, the medical humanities exchange underscored the potential for integrating cinemeducation into a broad spectrum of educational settings and for establishing a transdisciplinary cinemeducation symposium or film festival that fosters creative innovation and sustained transdisciplinary dialogue.

## Notes

### Author contributions

MR initiated the exchange. MR, SK, and JK developed the exchange concept. MR developed the teaching evaluation, performed the material preparation, data collection, and analysis, and wrote the first draft. All authors reviewed and revised subsequent manuscript versions and read and approved the final manuscript.

### Authors’ ORCIDs

- Mike Rueb: [0000-0002-2057-383X]
- Kevin B. Lee: [0000-0002-8701-0679]
- Martin R. Fischer: [0000-0002-5299-5025]

### Funding

The Locarno Film Festival and Locarno BaseCamp funded the exchange.

### Ethical approval and consent for publication

Ethics approval was not required as the project was a teaching evaluation.

## Competing interests

The authors declare that they have no competing interests. 

## Figures and Tables

**Table 1:**
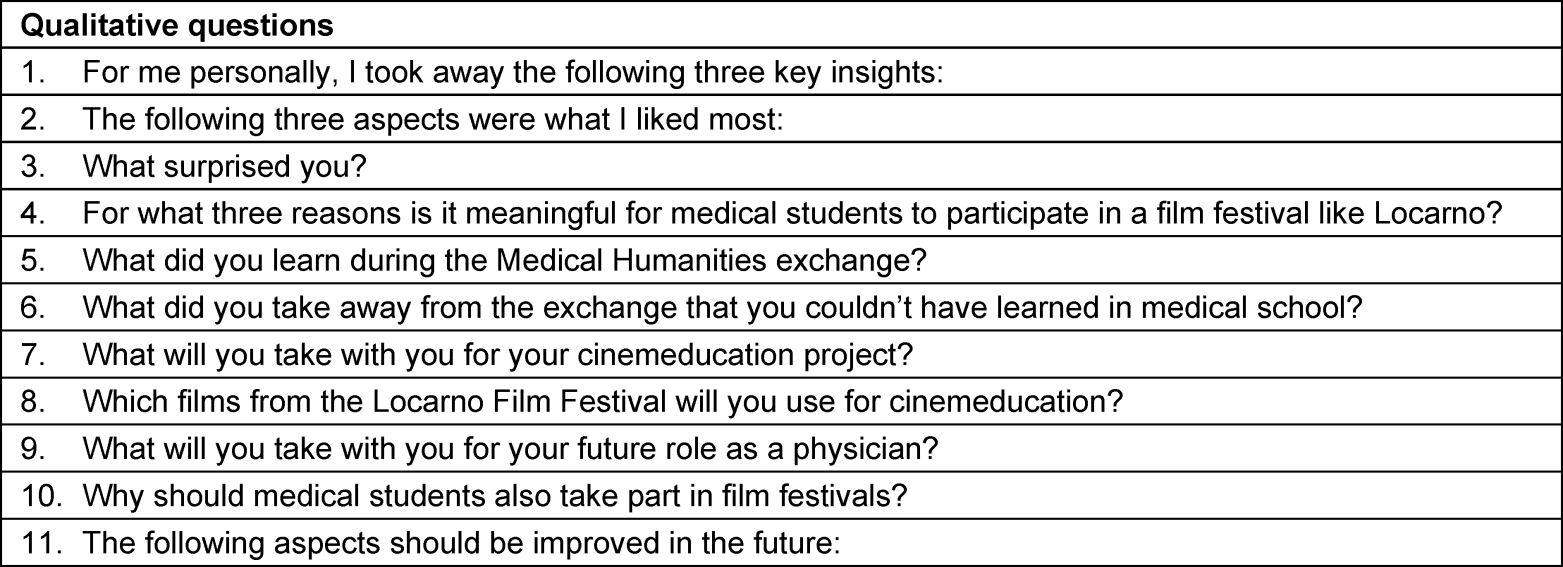
Qualitative questions for evaluating the transdisciplinary exchange

**Table 2:**
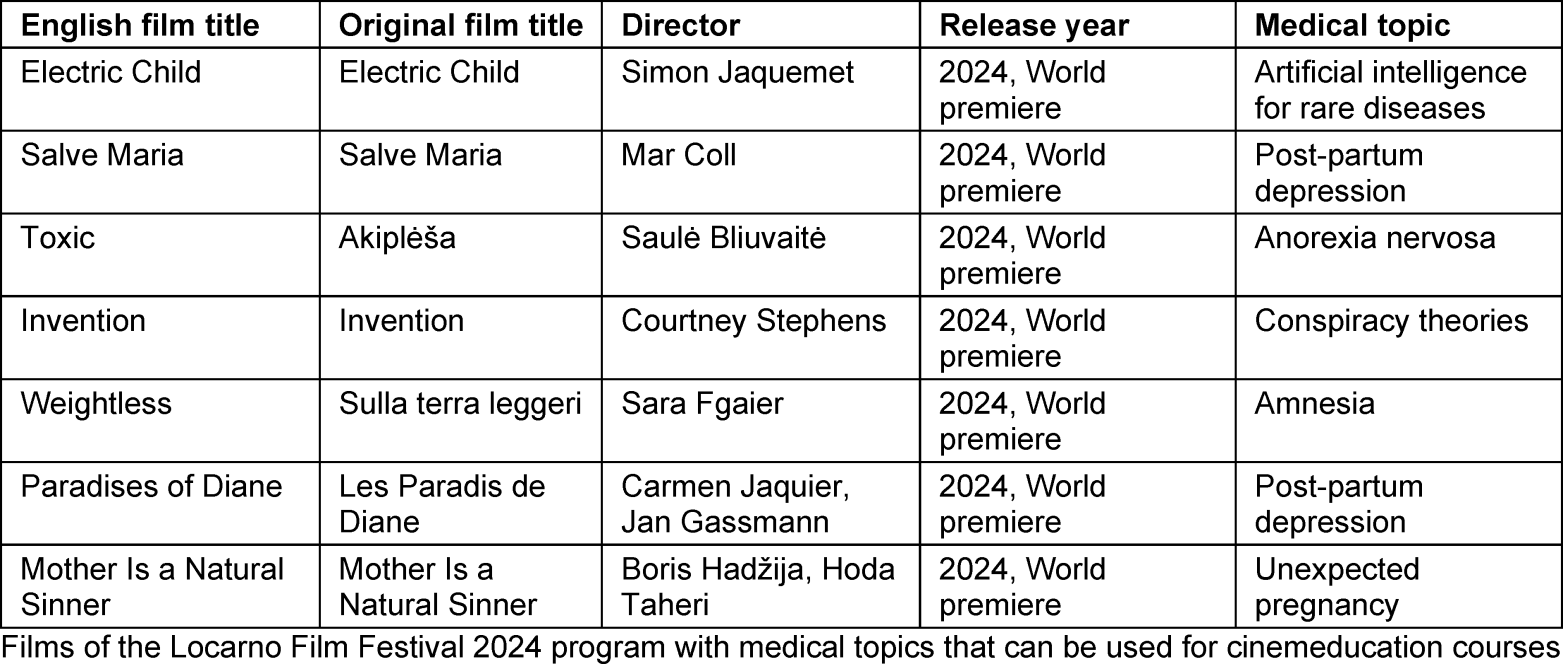
Films from the Locarno Film Festival for cinemeducation

**Table 3:**
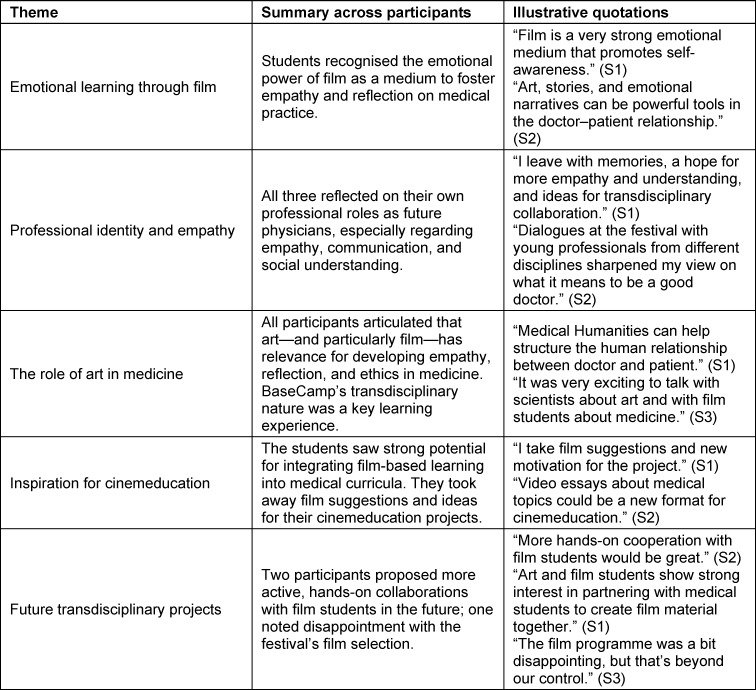
Qualitative evaluation of the medical humanities exchange

**Table 4:**
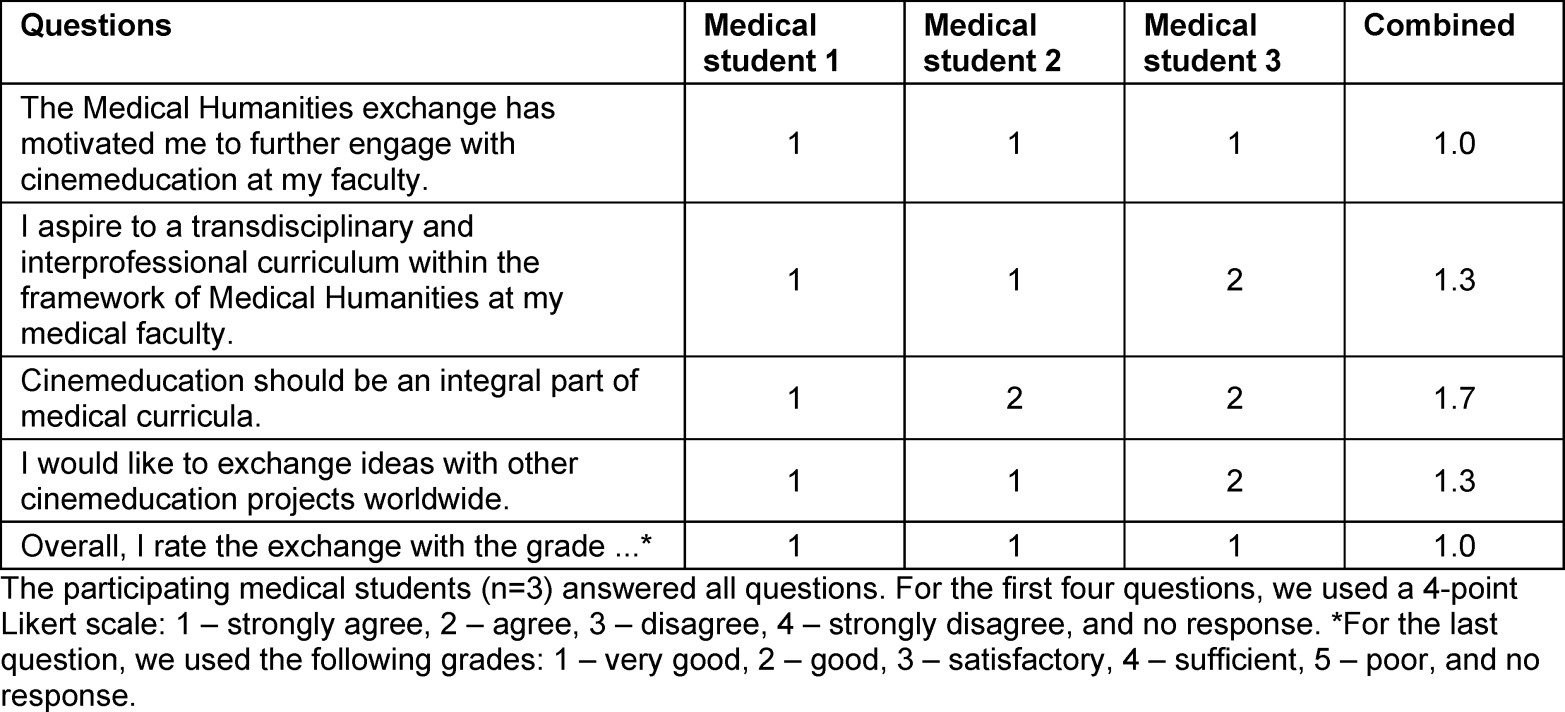
Quantitative evaluation of the medical humanities exchange
