# Erratum and republication: Contemporary cinemeducation: Transdisciplinary exchange at Locarno Film Festival

**DOI:** 10.3205/zma001864

**Published:** 2026-08-07

**Authors:** Mike Rueb, Stefano Knuchel, Justine Knuchel, Kevin B. Lee, Martin R. Fischer

**Affiliations:** 1 LMU Munich, LMU University Hospital, Institute of Medical Education, Munich, Germany; 2 Charité – Universitätsmedizin Berlin, Charité Campus Mitte, Department of Psychiatry and Neurosciences, Berlin, Germany; 3 Charité – Universitätsmedizin Berlin, Charité at St. Hedwig Hospital, Department of Psychiatry and Psychotherapy, Berlin, Germany; 4 Locarno Film Festival, BaseCamp, Locarno, Switzerland; 5 Università della Svizzera italiana, Locarno Film Festival Professor for the Future of Cinema and the Audiovisual Arts, Lugano, Switzerland; 6 Medical University of Vienna, Teaching Center, Vienna, Austria

 Erratum for: Rueb M, Knuchel S, Knuchel J, Lee KL, Fischer MR. Contemporary cinemeducation: Transdisciplinary exchange at Locarno Film Festival. GMS J Med Educ. 2026;43(5):Doc58. DOI: 10.3205/zma001852

## Notification

Following the publication of the article

Rueb M, Knuchel S, Knuchel J, Lee KL, Fischer MR. Contemporary cinemeducation: Transdisciplinary exchange at Locarno Film Festival. GMS J Med Educ. 2026;43(5):Doc58. [DOI: 10.3205/zma001852]

it was discovered that the author Kevin Lee’s middle name had been listed incorrectly. 

Therefore the article has been corrected and republished: 

Rueb M, Knuchel S, Knuchel J, Lee KB, Fischer MR. Contemporary cinemeducation: Transdisciplinary exchange at Locarno Film Festival. GMS J Med Educ. GMS J Med Educ. 2026;43(5):Doc71. [DOI: 10.3205/zma001865]

The author’s abbreviated middle name and his initials in the citation note have been corrected; no other changes have been made to the article.

## Authors’ ORCIDs

- Mike Rueb: [0000-0002-2057-383X]
- Kevin B. Lee: [0000-0002-8701-0679]
- Martin R. Fischer: [0000-0002-5299-5025]

## Competing interests

The authors declare that they have no competing interests. 

